# Information Thermodynamics Derives the Entropy Current of Cell Signal Transduction as a Model of a Binary Coding System

**DOI:** 10.3390/e20020145

**Published:** 2018-02-24

**Authors:** Tatsuaki Tsuruyama

**Affiliations:** Department of Discovery Medicine, Pathology Division, Graduate School of Medicine, Kyoto University, Yoshida-Konoe-cho, Sakyo-ku, Kyoto 606-8315, Japan; tsuruyam@kuhp.kyoto-u.ac.jp; Tel.: +81-75-753-4427; Fax: +81-75-753-4493

**Keywords:** signal transduction, fluctuation theorem, entropy production rate

## Abstract

The analysis of cellular signaling cascades based on information thermodynamics has recently developed considerably. A signaling cascade may be considered a binary code system consisting of two types of signaling molecules that carry biological information, phosphorylated active, and non-phosphorylated inactive forms. This study aims to evaluate the signal transduction step in cascades from the viewpoint of changes in mixing entropy. An increase in active forms may induce biological signal transduction through a mixing entropy change, which induces a chemical potential current in the signaling cascade. We applied the fluctuation theorem to calculate the chemical potential current and found that the average entropy production current is independent of the step in the whole cascade. As a result, the entropy current carrying signal transduction is defined by the entropy current mobility.

## 1. Introduction

The cell is an open and non-equilibrium system, and signal transduction is one of the non-equilibrium processes characterized by a chemical potential current. In recent decades, the theoretical analysis of signal transduction has been broadly applied in various research fields, in parallel with significant development of information theory [1,2,3,4,5]. Informational thermodynamics for analyzing dynamic biochemical networks and systems biology have also been developed to assess the cell response to external stimuli [1,2,3,4,5,6]. In addition, in in vivo analysis, a significant amount of data on signal transduction has accumulated, and the quantitative analysis of a network of signaling cascades can be performed using new technology [7,8,9,10,11,12,13,14,15,16]. In this study, a quantitative evaluation theory of a signaling cascade is described from the source coding theory of a binary code system applied for biological signal transduction. The ubiquitous signaling pathway conveys signals from the cell membrane to the nucleus shown as a form of a model scheme.

Let us consider a cell system as an open homogeneous reactor in contact with chemiostats of reactants and products, which drive the system out of equilibrium. The system is assumed to be isothermal and isovolumic.

In this model, the signaling molecule at step *j*, denoted as *X_j_*, induces the modification of *X_j_*_+1_ into *X_j_*_+1_*. In individual steps, the dephosphorylation of *X_j_*_+1_* into *X_j_*_+1_ occurs by phosphatase *Ph_j_* by the release ability of inorganic phosphate Pi from *X_j_*_+1_*, and the pre-stimulation steady state is subsequently recovered. A signaling step in the above cascades may be described as follows:

(1) $$\begin{array}{lll} {X_{j}}^{*}+X_{j+1}+\mathrm{ATP}\to {X_{j}}^{*}+{X_{j+1}}^{*}+\mathrm{ADP} & :k_{j} & \\ {X_{j+1}}^{*}+Ph_{j}\to X_{j+1}+Pi & :k_{-j} & \left(1\leq j\leq n\right) \end{array}$$

*k_j_* and *k_−j_* are the kinetic coefficients. ATP, ADP, and Pi represent adenosine triphosphate, adenosine diphosphate, and inorganic phosphate, respectively. Consider all the possible distinct signal transduction events that correspond to all the possible combinations of the signal molecule *X_j_*, whose transduction length is $\tau_{j}$. For instance, an event is described as follows:

*X*_1_ *X*_3_ *X*_2_ *X*_3_ *X*_1_ *X*_2_ *X*_3_ *X*_5_ *X*_3_ *X*_4_ *X*_3_ (2)

The cell signaling events, represented here by the symbols *X_j_* with numbers $N_{j}\left(1\leq j\leq n\right)$, will correspond to all the possible combinations of *X_j_*. Therefore, *N*_1_ = 2, *N*_2_ = 2, *N*_3_ = 5, *N*_4_ = 1, *N*_5_ = 1 and *n* = 5 in the signal event (2). In actuality, signaling cascades have been studied extensively using models of Mitogen-activated Protein Kinase (MAPK) pathways, in which the epidermal growth factor receptor, c-Raf, MAP kinase-extracellular signal-regulated kinase [17], and kinase-extracellular signal-regulated kinase (ERK) are phosphorylated following treatment with growth factors. The Ras-*c*-Raf-ERK cascade (RRE) is a ubiquitous signaling pathway that conveys mitogenic and differentiation signals from the cell membrane to the nucleus.

(3) $$\begin{array}{l} L+R↔R^{*},\;R^{*}+\mathrm{Ras}↔R^{*}+{\mathrm{Ras}}^{*}(X_{1}),\\ {\mathrm{Ras}}^{*}+c-\mathrm{Raf}↔c-{\mathrm{Raf}}^{*}\left(X_{2}\right)+{\mathrm{Ras}}^{*},\\ c-{\mathrm{Raf}}^{*}+\mathrm{MEK}↔c-{\mathrm{Raf}}^{*}+{\mathrm{MEK}}^{*},\\ {\mathrm{MEK}}^{*}+\mathrm{ERK}↔\mathrm{MEK}+{\mathrm{ERK}}^{*}\left(X_{3}\right) \end{array}$$

**Figure 1:**
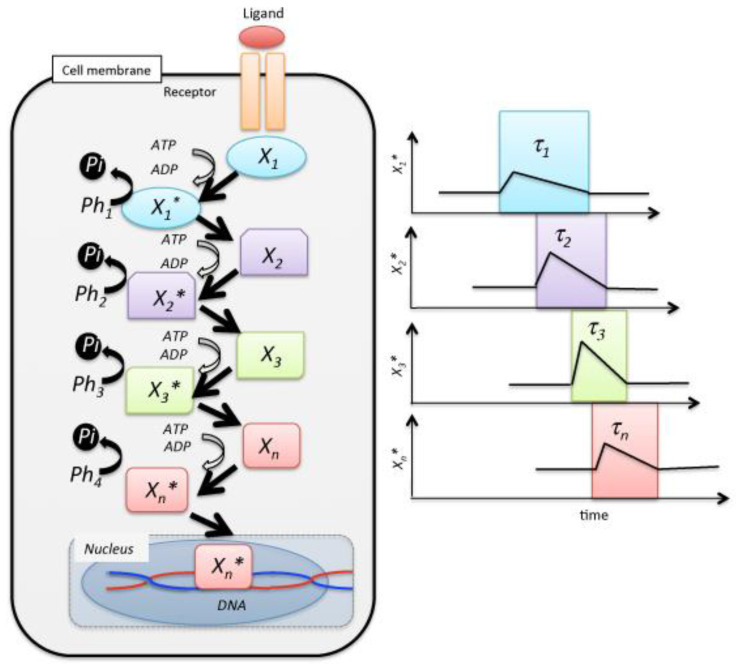
Schematic of a reaction cascade in cell signal transduction. The receptor mediates the cellular response to the presence of the ligand in the extracellular medium. A is a messenger, ATP, of signal transduction. Individual signaling molecules $X_{j}\left(1\leq j\leq n\right)$ relay the modification of individual steps, and the last species $X_{n}$ is translocated to the nucleus, where it controls gene expression by the transcription of mRNA. Ph denotes a phosphatase.

In the above equation, R and L represent the receptor in the cell membrane and the ligand that is substance stimulating receptor, respectively. External stimulation on the cell induces a concentration fluctuation in the phosphorylation of the signaling molecules. More specifically, a fluctuation in the signaling molecules’ concentration tentatively increases, followed by a decrease over a long duration, $\tau_{j}$, of several hours [7,8,9,10,11,12,13,14,15,16,17,18,19,20,21,22,23,24] (Figure 1). Here, we defined the occurrence probability, $p_{j}$ and ${p_{j}}^{*}$, which represents the selection probability of *j*-th step using *X_j_* or X_j_*, respectively:

(4) $$p_{j}=X_{j}/X$$

(5) $${p_{j}}^{*}={X_{j}}^{*}/X$$

with

(6) $$\sum_{j=1}^{n}p_{j}+{p_{j}}^{*}=1$$

Here, X represents the total concentration of signaling molecules.

(7) $$X=\sum_{j=1}^{n}X_{j}+{X_{j}}^{*}$$

Because the sum of the concentrations of active j molecules, X_j_*, and non-active j molecules, *X_j_*, participating in signaling cascades is regarded as constant, the protein production process is relatively slower than the signal transduction step:

(8) $$p_{j}+{p_{j}}^{*}=p_{j}{}^{0}$$

The entire duration, τ, which signifies the sum of cascades, is determined using the total concentration of signaling molecules, X, and the probabilities, $p_{j}$ and ${p_{j}}^{*}$.

Here, $\tau_{j}$ signifies the signal step duration of the *j*-step of the cascade. Subsequently, the total number of signal events, ψ, is introduced for the whole cascade, as follows:

(9) $$\psi =\frac{X!}{\prod_{j=1}^{n}X_{j}!\prod_{j=1}^{n}{X_{j}}^{*}!}$$

The logarithm of ψ, which is Shannon’s entropy *S*, is given using Stirling’s approximation, as follows:

(10) $$S=\log\psi ≃-k_{B}X\left(\sum_{j=1}^{n}p_{j}\log p_{j}+\sum_{j=1}^{n}{p_{j}}^{*}\log{p_{j}}^{*}\right)$$

In Equation (10), $k_{B}$ represents the Boltzmann constant.

## 2. Mixing Entropy in Signal Transduction 

Here, we noticed that the right side of Equation (10) is identical to mixing entropy in the system in which ${X_{j}}^{*}$ and $X_{j}$ are mixed. Here, our aim was to estimate the entropy change at individual steps in the cascade. Because the signaling molecules $X_{j}$ are macromolecules, they are localized and the individual steps are compartmentalized in the in the cytoplasm. In the steady state, the signal transduction system stands at steady state. Here, let us consider that the ligand molecule stimulates the given system, and this stimulation produces a fluctuation in the transduction system. Considering the entropy current from the *j*-th to (*j* + 1)-th step by the mixing entropy consisting of active molecule X_j_* and *X_j_* difference between the steps, the mixing entropy change of the *j*-th step, $dS_{j}^{\mathrm{mix}}$, with a minimal concentration difference in X_j_*, $d{p_{j}}^{*}$, and in *X_j_*, $dp_{j}=-d{p_{j}}^{*}$, is described:

(11) $$dS_{j}^{\mathrm{mix}}≜-k_{B}X\left[\left(p_{j}+dp_{j}\right)\log\left(p_{j}+dp_{j}\right)+\left({p_{j}}^{*}+d{p_{j}}^{*}\right)\log\left({p_{j}}^{*}+d{p_{j}}^{*}\right)\right]$$

In Equation (11), $d{p_{j}}^{*}$ and $dp_{j}$ denote the fluctuations in the transduction system. On the other hand, because the increase and the decrease are not observed in the (*j* + 1)-th step in the initial phase of the signal transduction from the *j*-th to (*j* + 1)-th step:

(12) $$dS_{j+1}^{\mathrm{mix}}≜-k_{B}X\left[p_{j}\log p_{j}+{p_{j}}^{*}\log{p_{j}}^{*}\right]$$

Here, T represents the temperature of the given system. Then, we have the entropy signal current $C_{j}$ arising from chemical potential difference on the left side of Equations (11) and (12) using differential coefficient of missing entropy for ${p_{j}}^{*}$, which is the probability of selection of an active molecule that transmits the signal transduction:

(13) $$C_{j}=T\frac{\partial dS_{j}^{\mathrm{mix}}}{\partial {p_{j}}^{*}}\Delta {p_{j}}^{*}\approx k_{B}TX\log\frac{p_{j}}{{p_{j}}^{*}}\Delta {p_{j}}^{*}=k_{B}T\log\frac{p_{j}}{{p_{j}}^{*}}\Delta {X_{j}}^{*}$$

Therefore, the entropy signal current density $c_{j}$ from the entropy change is given:

(14) $$c_{j}=\frac{C_{j}}{\Delta X_{j}}=k_{B}T\log\frac{p_{j}}{{p_{j}}^{*}}$$

In general, such a nonequilibrium steady system is given by the occurrence probability *p* during signal step duration $\tau_{j}$ for the current $c_{j}$ from the left reservoir at temperature $\beta_{L}{}^{-1}$ and chemical potential $\mu_{L}$ to the right reservoir at $\beta_{L}{}^{-1}$ and $\mu_{R}$ satisfies the steady fluctuation theorem [25]:

(15) $$\underset{\tau_{j}\to \infty }{\lim}\frac{1}{\tau_{j}}\log\left\{\frac{p\left(j+1|j;{q_{j}}^{*}\right)}{p\left(j|j+1;-{q_{j}}^{*}\right)}\right\}=\frac{\beta_{L}\mu_{L}-\beta_{R}\mu_{R}}{\tau_{j}}{q_{j}}^{*}$$

Here, $q_{j}$ represents the flow of the signal current.

This fluctuation theorem leads to various nonequilibrium relations among cumulates of the current. Because in the biological systems, $\beta_{L}{}^{-1}=\beta_{R}{}^{-1}=\beta^{-1}$ and using $\beta^{-1}=k_{B}T$, we have using signal current density:

(16) $$\underset{\tau_{j}\to \infty }{\lim}\frac{1}{\tau_{j}}\log\frac{p\left(j+1|j\right)}{p\left(j|j+1\right)}=\frac{c_{j}}{k_{B}T\tau_{j}}\Delta {X_{j}}^{*}$$

On the left side of (16), p(j+1|j), the transitional probability of step j+1 given step j, is defined in the forward direction of the signal is also defined. From Equations (15) and (16) we have an important result:

(17) $$\underset{\tau_{j}\to \infty }{\lim}\frac{1}{\tau_{j}}\log\frac{p\left(j+1|j\right)}{p\left(j|j+1\right)}=\frac{1}{\tau_{j}}\log\frac{p_{j}}{{p_{j}}^{*}}$$

Equation (17) shows the relation between the transitional probability and the occurrence probability. In most cases of biological signal transduction, the signal duration is sufficiently long, and therefore Equation (17) can be further described simply as follows:

(18) $$\log\frac{p\left(j+1|j\right)}{p\left(j|j+1\right)}=\log\frac{p_{j}}{{p_{j}}^{*}}$$

In our previous reports [26,27], when the number of the events or messages per a given duration is maximized, the occurrence probability can be described using an arbitrary parameter ζ independent of the step number j:

(19) $$-\log p_{j}=\zeta \tau_{j}$$

Further, as shown in Appendix A, Equation (18) can be rewritten as follows:

(20) $$\frac{1}{\tau_{j}}\log\frac{p\left(j+1|j\right)}{p\left(j|j+1\right)}=-\zeta$$

As a result, we have an important result from Equations (16) and (20):

(21) $$\zeta =-\frac{c_{j}\Delta {X_{j}}^{*}}{k_{B}T\tau_{j}}=-\frac{C_{j}}{k_{B}T\tau_{j}}=-\frac{J}{k_{B}T}$$

with

(22) $$J≜\frac{C_{j}}{\tau_{j}}=k_{B}T\zeta$$

Here, J represents the average entropy production current along the cascade, and the suffix j representing the step number is omitted because the average current is independent of the step number. The above equation represents that ζ has the dimension of the average entropy production rate.

## 3. Entropy Current and Signal Transduction

Subsequently, we aimed to formulate the signal current from the perspectives of the entropy current. The entropy current depends on the spatial gradient of entropy and is given using the signal current intensity from (13) and (22) using a intracellular spatial coordinate parameter r:

(23) $$-T\nabla_{r}S_{j}\approx -k_{B}T\log\frac{p_{j}}{{p_{j}}^{*}}\nabla_{r}{X_{j}}^{*}=-c_{j}\nabla_{r}{X_{j}}^{*}=-\nabla_{r}C_{j}$$

Further, using the diffusion coefficient of an active signaling molecule, the average entropy current per signal duration is given using the diffusion coefficient of the signal, $D_{j}$ and from (22):

(24) $$\nabla_{r}J=-\frac{D_{j}\nabla_{r}{X_{j}}^{*}}{\tau_{j}}=-\frac{c_{j}\nabla_{r}{X_{j}}^{*}}{\tau_{j}}$$

Here, the diffusion coefficient is obtained from (23):

(25) $$D_{j}≜c_{j}$$

From the Stokes–Einstein equation, the diffusion coefficients can be described using the signal mobility, $\omega_{j}$:

(26) $$D_{j}≜k_{B}T\omega_{j}$$

Therefore, we have:

(27) $$c_{j}=k_{B}T\omega_{j}$$

## 4. Conclusions

In the current study, we hypothesized that the signaling cascade is a binary code system in which an increase in the concentration of the active signal molecule at each step (although accompanied by a decrease in the inactive form of the signal molecule) transmits a signal transduction between steps. This simple binary-code hypothesis enabled us to have several important equities about the quantification of signal transformation. This hypothesis includes a theoretical basis that can introduce a duration parameter to analyze the development of signal transduction over time. We postulate that quantitative estimation of signal transduction is possible based on the amount of ATP consumed. In an actual experiment, preventing other biochemical reactions, apart from signal transduction, is challenging, but we have a plan for establishing a model for measurement. Therefore, if data with adequate comprehensive signal events is accumulated, quantification of the signal events might be possible by measurement of ATP concentration changes.

According to this idea and the fluctuation theorem [28,29,30], we obtained an important result: the diffusion coefficient of the signal event is equal to the entropy current. In this way, the signal transduction in the cell system is definitely formulated in the entropy production and the current. Significantly, the average entropy production rate current is independent of the step number; this implies that the whole cascade of the signal transduction is integrated well under stable entropy production. Based on this finding, the quantification of the signal events is possible by measuring the chemical potential change during individual signal event in the cell system. For example, the consumption of ATP, which mediates signal transduction, is anticipated to provide data regarding the entropy production during the events. In conclusion, the current signaling cascade model provides a basis for informational thermodynamics, and the relationship between the chemical potential and information or entropy was established.

## Appendix A

To obtain Equation (20) from Equations (8), (18) and (19), we can calculate as follows:

$$\begin{array}{l} \frac{1}{\tau_{j}}\log\frac{p_{j}}{{p_{j}}^{*}}=\frac{1}{\tau_{j}}\log\frac{\exp\left(-\zeta \tau_{j}\right)}{p_{j}^{0}-\exp\left(-\zeta \tau_{j}\right)}=\frac{1}{\tau_{j}}\log\frac{\exp\left(-\zeta \tau_{j}\right)}{p_{j}^{0}\left[1-\exp\left(-\zeta \tau_{j}\right)/p_{j}^{0}\right]}\\ =-\zeta -\frac{1}{\tau_{j}}\log p_{j}^{0}-\frac{1}{\tau_{j}}\log\left[1-\exp\left(-\zeta \tau_{j}\right)/p_{j}^{0}\right]\\ =-\zeta -\frac{1}{\tau_{j}}\log p_{j}^{0}+\frac{1}{\tau_{j}}\exp\left(-\zeta \tau_{j}\right)/p_{j}^{0}\\ \approx -\zeta \left(\tau_{j}\to \infty \right) \end{array}$$
